# The Impact of Cell-Expansion and Inflammation on The Immune-Biology of Human Adipose Tissue-Derived Mesenchymal Stromal Cells

**DOI:** 10.3390/jcm9030696

**Published:** 2020-03-04

**Authors:** Karolien Buyl, Makram Merimi, Robim M. Rodrigues, Douâa Moussa Agha, Rahma Melki, Tamara Vanhaecke, Dominique Bron, Philippe Lewalle, Nathalie Meuleman, Hassan Fahmi, Vera Rogiers, Laurence Lagneaux, Joery De Kock, Mehdi Najar

**Affiliations:** 1 Department of In Vitro Toxicology and Dermato-Cosmetology, Vrije Universiteit Brussel (VUB), Laarbeeklaan 103, 1090 Brussels, Belgium; 2 Laboratory of Experimental Hematology, Institut Jules Bordet, Université Libre de Bruxelles (ULB), 121 Boulevard de Waterloo, 1000 Bruxelles, Belgium; 3 Genetics and Immune Cell Therapy Unit, Faculty of Sciences, University Mohammed Premier, Oujda 60000, Morocco; 4 Osteoarthritis Research Unit, University of Montreal Hospital Research Center (CRCHUM), 900 Saint-Denis, R11.424, Montreal, QC H2X 0A9, Canada; 5 Laboratory of Clinical Cell Therapy, Institut Jules Bordet, Université Libre de Bruxelles (ULB), 808 Route de Lennik, 1070 Brussels, Belgium

**Keywords:** cellular therapy, adipose tissue, mesenchymal stromal cells, immune-biology, screen, inflammation, in vitro expansion, tissue repair

## Abstract

**Background:** As a cell-based therapeutic, AT-MSCs need to create an immuno-reparative environment appropriate for tissue repair. In the presence of injury, MSCs may have to proliferate and face inflammation. Clinical application requires repeated administrations of a high number of cells with a well-established immune profile. **Methods:** We have established an immuno-comparative screening by determining the expression of 28 molecules implicated in immune regulation. This screening was performed during cell-expansion and inflammatory priming of AT-MSCs. **Results:** Our study confirms that AT-MSCs are highly expandable and sensitive to inflammation. Both conditions have substantially modulated the expression of a panel of immunological marker. Specifically, CD34 expression was substantially decreased upon cell-passaging. HLA-ABC, CD40 CD54, CD106, CD274 and CD112 were significantly increased by inflammation. In vitro cell-expansion also significantly altered the expression profile of HLA-DR, CD40, CD62L, CD106, CD166, HLA-G, CD200, HO-1, CD155 and ULBP-3. **Conclusion:** This study points out the response and characteristics of MSCs following expansion and inflammatory priming. It will strength our knowledge about the molecular mechanisms that may improve or hamper the therapeutic potential of MSCs. These immunological changes need to be further characterized to guarantee a safe cellular product with consistent quality and high therapeutic efficacy.

## 1. Introduction

The use of mesenchymal stromal cells (MSCs) as a cellular therapeutic product is actually harboring relevant perspectives and challenges. MSCs are undifferentiated, multipotent postnatal cells that reside within their niche between differentiated cells of the specific organ or tissue [1]. Normally they are found in a quiescent state and they will be activated in the case of disease or tissue damage, in order to repair the affected organ(s) or tissue(s) [2]. MSCs are defined by their self-renewal and in vitro differentiation potential. They also have a favorable phenotype and immunoregulatory profile. Therefore, their therapeutic potential is explored for cell-based therapies going from tissue repair, over regenerative medicine to immunomodulation [3,4,5]. MSCs reside within several human tissues, including the bone marrow, the skin, the umbilical cord and adipose tissue. When isolated for therapeutic purposes, important parameters should be considered, such as accessibility of the organ/tissue, the safety of the collection procedure and the amount of MSCs that can be isolated. Adipose tissue (AT) represents an ideal source for MSC isolation, as it is ubiquitously present in every human, and high numbers of MSCs can be harvested by using a minimally invasive procedure (liposuction) [6,7]. Moreover, some factors are believed to be relevant regarding the therapeutic success of using cultured MSCs: targeting the right donor population, addressing the optimal culture conditions and selecting the patient population that is most likely to give a positive therapeutic response [8].

MSCs, including AT-MSCs, are often investigated as cellular therapeutic product for managing diseases in which different environmental signals, such as inflammation, contribute to tissue damage [9]. Inflammation is likely to regulate several features of stem cells and thus to play an important role in health and disease [10]. Importantly, it can shape stem cells and stemness during infection and beyond. Furthermore, clinical applications of MSCs demand repeated administrations and large amounts of cells. This implies in vitro expansion of the obtained AT-MSCs throughout serial passaging [11]. Accordingly, the safety and efficiency of AT-MSCs as therapeutic products are closely related to their profile. We previously indicated that some immunobiological criteria that define MSCs may contribute to reach the right therapeutic issue [12]. The expression and modulation of molecules belonging to the Human Leukocyte Antigen (HLA) family, co-stimulatory pathway, cell–cell interaction pattern and immunoregulatory factors are of importance in governing the immunological behavior of MSCs [13]. Several immune-related molecules are involved in graft rejection and tissue repair. Recognition of foreign HLA class I and II molecules are critical for the graft-versus-host disease (GVHD) and allograft rejection. They operate as primary targets of the immune response and play a role in lymphocytes’ activation throughout co-stimulatory molecules [11,14,15]. It is now recognized that the communication of AT-MSCs with the environment is an essential part of their tissue repair process. MSCs can actively sense their surroundings and modulate, accordingly, their fate and behavior. In the presence of injury, AT-MSCs may have to proliferate (cell-expansion), as well as respond to inflammatory signals (inflammation), in order to create an immuno-reparative microenvironment. It is therefore important to understand the effect of cell-expansion and inflammation on the immune-biology of MSCs, in order to maximize their beneficial outcomes. In comparison to previous studies, this is the first time that these two relevant features of MSCs are explored and, most importantly, investigated in combination. The immunological profile of the AT-MSCs, obtained by Ficoll gradient centrifugation, was evaluated by flow cytometric analyses, under these two conditions. The expression of cell adhesion molecules, immune regulatory molecules and natural killer ligands were thus investigated. A detailed characterization of AT-MSCs under these conditions, with 28 examined markers, is therefore presented. Several changes and alterations within the expression profiles of these molecules are observed during cell-expansion and inflammation. Such an analysis will greatly help us to understand the molecular mechanisms that may improve or hamper the therapeutic potential of AT-MSCs, contributing thus to the efforts in developing a quality, safe and efficient cellular-therapeutic product.

## 2. Materials and Methods

### 2.1. Adipose Tissue Collection

After the patients signed informed consent forms, we collected lipoaspirate from male and female patients (age range 26–46 [36 ± 9] years) undergoing elective liposuction. The procedure was run in cooperation with the Department of Plastic Surgery of the UZ-Brussels (Brussels, Belgium) and the ATLAS clinic (Brussels, Belgium).

### 2.2. Isolation and Cultivation of AT-MSCs

AT-MSCs were isolated from 6 patients, as previously reported [16]. In brief, 125 mL of liposuction material was extensively washed by centrifugation (3 min at 600 g), using equal volumes of phosphate-buffered saline (PBS) to remove erythrocytes. The samples were incubated for 45 min, at 37 °C, with dissociation medium (1:1), that is, 125 mL aliquots of fat + 125 mL of dissociation medium. The latter consists of 1% (v/v) bovine serum albumin (BSA) (Sigma-Aldrich, Diegem, Belgium) and 1 mg/mL collagenase A (Roche Applied Science, Vilvoorde, Belgium) in PBS. The digested tissue was then passed through a mesh filter, to remove connective tissue debris. Subsequently, the filtrate was centrifuged for 10 min, at 600 g (4 °C), and the supernatant is removed. In the classical Ficoll gradient protocol, the cells were suspended in 50 mL of PBS supplemented with 1% (v/v) BSA and centrifuged again for 10 min, at 600 g (4 °C), after which the pellet was resuspended in 30 mL of PBS and supplemented with 1% (v/v) BSA. The cell suspension was then carefully brought on top of 15 mL of Ficoll gradient solution (Sigma-Aldrich) and centrifuged for 20 min, at 1000 g (4 °C). Upon centrifugation, the top layer was removed and the AT-MSCs were collected in 50 mL of PBS supplemented with 1% (v/v) BSA. The cell suspension was centrifuged for 10 min, at 600 g (8 °C), after which the supernatant was removed. The cell pellet was cultured in a 58 cm^2^ Petri dish (Greiner Bio One, Vilvoorde, Belgium) in Dulbecco’s Modified Eagle Medium (Lonza, Braine-1′Alleud, Belgium) supplemented with 10% (v/v) fetal bovine serum (HyClone, Perbio Science, Erembodegem-Aalst, Belgium), 7.33 IU/mL of benzyl penicillin (Continental Pharma, Brussels, Belgium), 50 μg/mL of streptomycin sulphate (Sigma-Aldrich) and 2.5 μg/mL of Fungizone (Invitrogen, Merelbeke, Belgium). This stage is referred to as the primary culture (PM). After 5 days of culture at 37 °C, under an atmosphere of 5% CO_2_ and 95% air, non-adherent cells were removed by replacing the medium. Throughout the culture period, the growth medium was changed once a week. Culture was pursued until 90% confluence was reached. Then, the cells were harvested, using TrypLE Select, and counted, using a 0.4% (w/v) trypan blue dye solution (Sigma-Aldrich). The cells were replated at a low-density of 1000 cells/cm^2^ (= passage 1). Cell populations were expanded and cultured in a similar way, until passage (P) 4.

### 2.3. Pro-Inflammatory Stimulation

The influence of an inflammatory signal on AT-MSCs was performed as described before [17]. Briefly, AT-MSCs were incubated for 18 h, with a pro-inflammatory cytokine cocktail containing 25 ng/mL of interleukin (IL)-1β (Peprotech, Rocky Hill, NJ, USA), 50 ng/mL of tumor necrosis factor (TNF)-α, 3000 U/mL or 10 ng/mL of interferon (IFN)-α and 1000 U/mL or 50 ng/mL of IFN-γ (all from Prospec Inc., Rehovot, Israel). After that, the medium was removed, and the cells were washed so they became available for further culture or analysis.

### 2.4. Flow Cytometric Analysis

The expression profile of 28 molecules was addressed by flow cytometry on a MacsQuant analyzer (MiltenyiBiotec, GmbH, Bergisch, Germany), using fluorochrome labeled monoclonal antibodies (Supplementary Table S1), as previously described [18]. Both the percentage (%) of positive cells and the mean fluorescence intensity (MFI) of each molecule were determined. The MFI represents the amount of each marker per cell that is present within the population.

### 2.5. Statistical Analysis

For statistical comparison of the flow cytometric analyses concerning the immunophenotype of the normal versus the pro-inflammatory conditions and the effect of in vitro expansion, a two-way ANOVA with Bonferroni’s post hoc test for multiple comparisons was performed. A *p*-value less than or equal to 0.05 is considered statistically significant (Prism v5.0d, Graph-Pad Software, USA).

## 3. Results

The phenotype of AT-MSCs was assessed by examining the constitutive expression of well-known immunological surface markers and immune regulatory molecules during in vitro expansion (PM-P1-P2-P3-P4), in the presence and absence of a pro-inflammatory signal. The data are presented as the mean ± SEM for each investigated marker in Supplementary Table S2 (percentage positive cells) and Supplementary Table S3 (MFI). These tables contain the data obtained for each passage (PM-P4) in the normal and the pro-inflammatory conditions.

### 3.1. Hematopoietic and Stromal Markers

**Figure 1:**
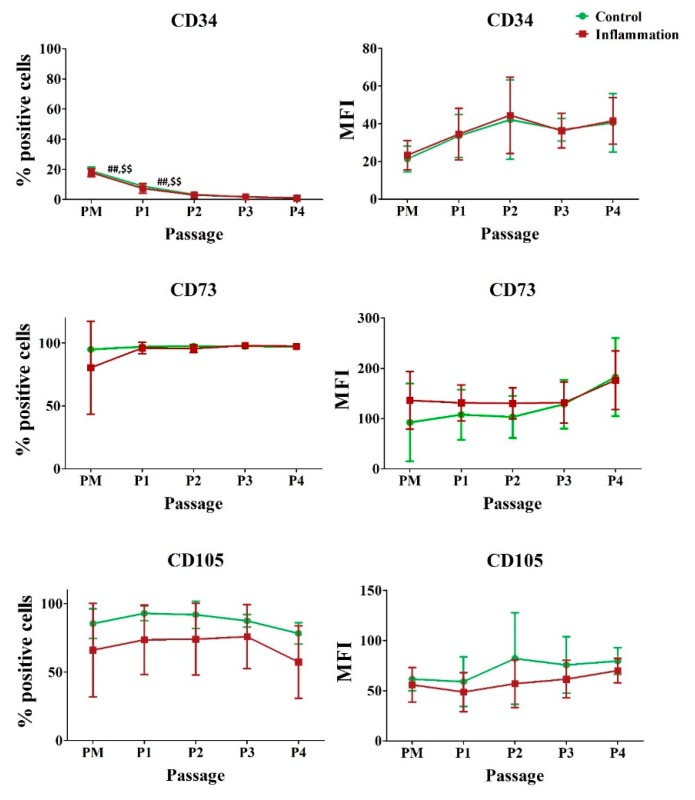
Impact of inflammation and cell passaging on the expression of endothelial/stromal markers in AT-MSCs. Flow cytometric analysis was used to determine the percentage of positive cells and the protein expression per cell (MFI). The values are expressed as mean ± SD and originate from six different AT-MSC donors. ^##^ Significantly lower percentage or amount per cell in non-stimulated AT-MSCs compared to the consecutive passage (*p*-value < 0.05). ^$$^ Significantly lower percentage or amount per cell in inflammation-stimulated AT-MSCs compared to the consecutive passage (*p*-value < 0.05).

AT-MSCs are reported to be positive for the hematopoietic stem cell marker cluster of differentiation (CD) 34. Such expression is likely to decrease during culture, until its disappearance [19]. We confirmed that the percentage of AT-MSCs expressing CD34 significantly decreases during in vitro expansion, both in normal and pro-inflammatory conditions. However, no significant differences could be detected in the amount of CD34 molecules expressed per cell. Furthermore, it was observed that, neither inflammation nor in vitro expansion has an impact on the expression of the stromal markers CD73 and CD105 (Figure 1; Supplementary Tables S2 and S3).

### 3.2. Human Leukocyte Antigens

**Figure 2:**
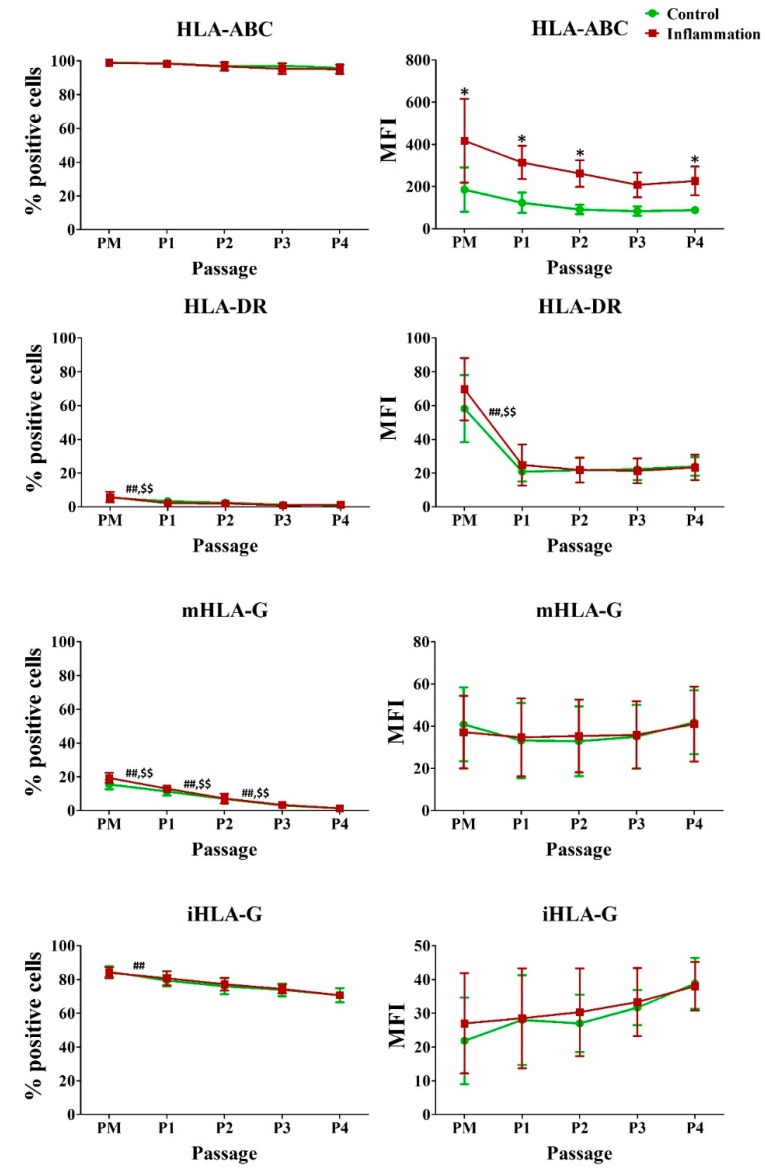
Impact of inflammation and cell passaging on the expression of human leukocyte antigens in AT-MSCs. Flow cytometric analysis was used to determine the percentage of positive cells and the protein expression per cell (MFI). The values are expressed as mean ± SD and originate from six different AT-MSC donors. ^*^ Significantly higher percentage or amount per cell in inflammation-stimulated AT-MSCs compared to regular AT-MSCs (*p*-value < 0.05). ^##^ Significantly lower percentage or amount per cell in regular AT-MSCs compared to the consecutive passage (*p*-value < 0.05). ^$$^ Significantly lower percentage or amount per cell in inflammation-stimulated AT-MSCs compared to the consecutive passage (*p*-value < 0.05).

Under normal conditions, AT-MSCs express HLA-ABC and intracellular (i) HLA-G, whilst a very-low to low expression is seen for HLA-DR and membrane bound (m) HLA-G, respectively. In vitro expansion of AT-MSCs significantly decreases the expression of HLA-DR, mHLA-G and iHLA-G. Indeed, for HLA-DR and iHLA-G, this is observed during the first passage only, whereas for mHLA-G, the expression further decreases for two more consecutive passages. For both HLA-DR and mHLA-G, this observation is also true in the pro-inflammatory conditions. In addition, pro-inflammatory stimulation significantly increases the expression of HLA-ABC per cell in AT-MSCs over the entire in vitro expansion period (Figure 2; Supplementary Tables S2 and S3).

### 3.3. Co-Stimulatory Molecules

**Figure 3:**
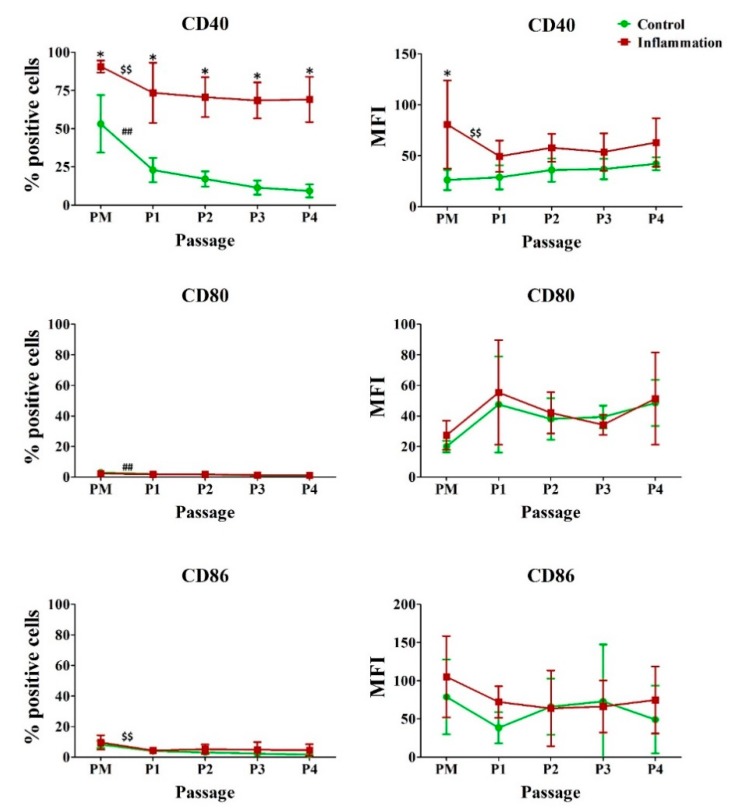
Impact of inflammation and cell passaging on the expression of co-stimulatory molecules in AT-MSCs. Flow cytometric analysis was used to determine the percentage of positive cells and the protein expression per cell (MFI). The values are expressed as mean ± SD and originate from six different AT-MSC donors. ^*^ Significantly higher percentage or amount per cell in inflammation-stimulated AT-MSCs compared to regular AT-MSCs (*p*-value < 0.05). ^##^ Significantly lower percentage or amount per cell in regular AT-MSCs compared to the consecutive passage (*p*-value < 0.05). ^$$^ Significantly lower percentage or amount per cell in inflammation-stimulated AT-MSCs compared to the consecutive passage (*p*-value < 0.05).

Under normal conditions, the co-stimulatory molecules CD80, CD86, CD134 and CD252 are only expressed by a low to very low percentage of AT-MSCs, whereas approximately 50% of AT-MSCs express CD40. Inflammation specifically affects the expression of the co-stimulatory molecule CD40 because a significant upregulation is detected for all the consecutive passages upon priming of AT-MSCs with inflammation. Moreover, for PM, a significant increase is observed upon pro-inflammatory stimulation for the amount of CD40 that is expressed per cell. Furthermore, in vitro expansion of AT-MSCs significantly decreases the expression of CD40 and CD80, and CD40 and CD86, respectively, in normal and pro-inflammatory conditions. No significant changes in CD134 and CD252 expression could be observed (Figure 3; Supplementary Tables S2 and S3).

### 3.4. Cell Adhesion Molecules

**Figure 4:**
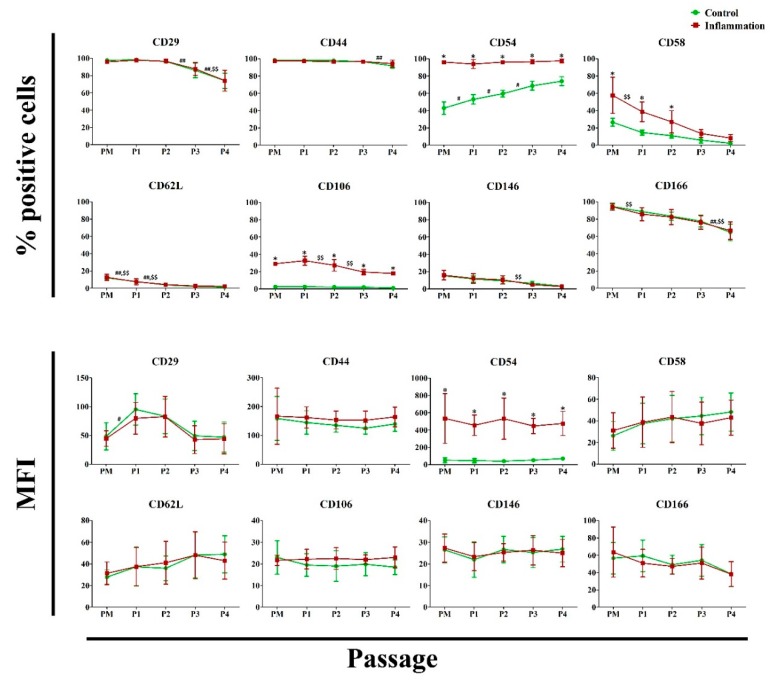
Impact of inflammation and cell passaging on the expression of cell adhesion molecules in AT-MSCs. Flow cytometric analysis was used to determine the percentage of positive cells and the protein expression per cell (MFI). The values are expressed as mean ± SD and originate from six different AT-MSC donors. ^*^ Significantly higher percentage or amount per cell in inflammation-stimulated AT-MSCs compared to regular AT-MSCs (*p*-value < 0.05). ^#^ Significantly higher percentage or amount per cell in regular AT-MSCs compared to the consecutive passage (*p*-value < 0.05). ^##^ Significantly lower percentage or amount per cell in regular AT-MSCs compared to the consecutive passage (*p*-value < 0.05). ^$$^ Significantly lower percentage or amount per cell in inflammation-stimulated AT-MSCs compared to the consecutive passage (*p*-value < 0.05).

Concerning the cell adhesion molecules, a significantly higher percentage of cells expressing CD54, CD58 and CD106 was detected during the in vitro expansion, when the AT-MSCs were exposed to a pro-inflammatory cytokine cocktail, as compared to the control conditions. For CD54, this is also observed for the amount of protein expressed per cell. Under normal conditions, the percentage of AT-MSCs expressing CD54 significantly increased during the in vitro expansion, whereas the percentage of cells expressing CD29, CD44, CD62L and CD166 significantly decreased. Similar results were also obtained under pro-inflammatory conditions, where the percentage of AT-MSCs expressing CD29, CD58, CD62L, CD106, CD146 and CD166 significantly decreased over one or more consecutive passages during the in vitro expansion period (Figure 4; Supplementary Tables S2 and S3). No significant changes in CD49e and CD102 expression could be noted (Supplementary Tables S2 and S3).

### 3.5. Immunoregulatory Molecules

**Figure 5:**
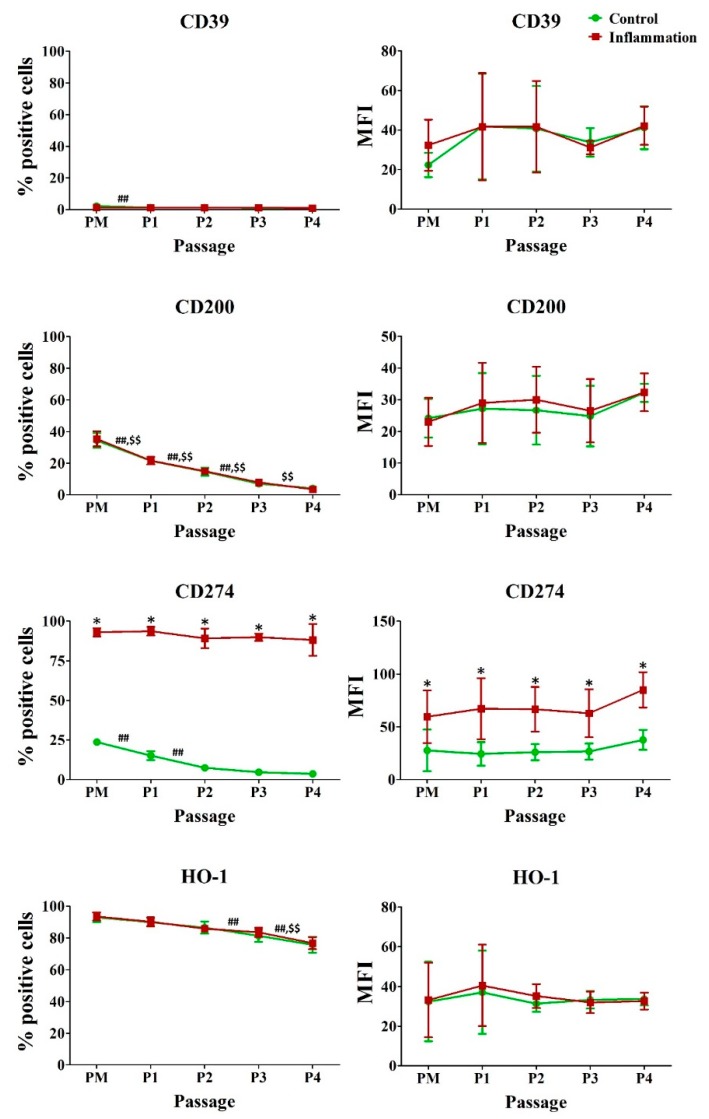
Impact of inflammation and cell passaging on the expression of immunoregulatory molecules in AT-MSCs. Flow cytometric analysis was used to determine the percentage of positive cells and the protein expression per cell (MFI). The values are expressed as mean ± SD and originate from six different AT-MSC donors. ^*^ Significantly higher percentage or amount per cell in inflammation-stimulated AT-MSCs compared to regular AT-MSCs (*p*-value < 0.05). ^##^ Significantly lower percentage or amount per cell in regular AT-MSCs compared to the consecutive passage (*p*-value < 0.05). ^$$^ Significantly lower percentage or amount per cell in inflammation-stimulated AT-MSCs compared to the consecutive passage (*p*-value < 0.05).

Under normal conditions, a significant percentage of AT-MSCs expressed CD200, CD274 and heme oxygenase (HO)-1, but their expression significantly decreased during in vitro expansion. This is also observed for CD200 and HO-1 in the pro-inflammatory condition. On the other hand, CD39 was only expressed by a very low percentage of AT-MSCs. Upon pro-inflammatory stimulation, the percentage of cells expressing CD274 significantly increased by approximately threefold, reaching around 90% of the cell population. A significant twofold increase was also observed for the amount of CD274 proteins expressed per cell (Figure 5; Supplementary Tables S2 and S3).

### 3.6. Natural Killer (NK) Ligands

**Figure 6:**
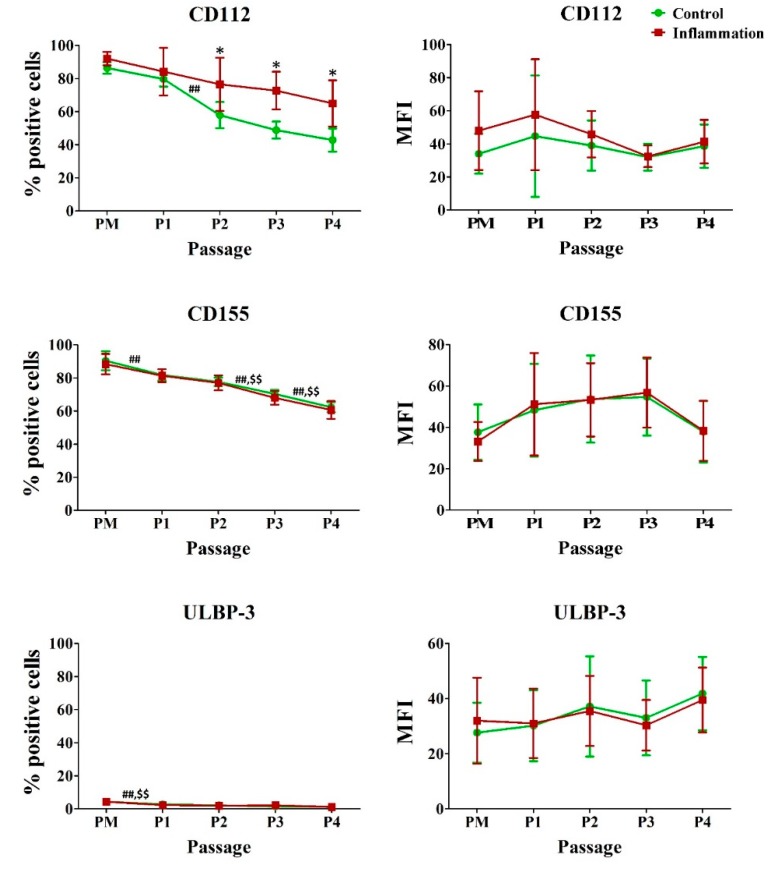
Impact of inflammation and cell passaging on the expression of natural killer ligands in AT-MSCs. Flow cytometric analysis was used to determine the percentage of positive cells and the protein expression per cell (MFI). The values are expressed as mean ± SD and originate from six different AT-MSC donors. ^*^ Significantly higher percentage or amount per cell in inflammation-stimulated AT-MSCs compared to regular AT-MSCs (*p*-value < 0.05). ^##^ Significantly lower percentage or amount per cell in regular AT-MSCs compared to the consecutive passage (*p*-value < 0.05). ^$$^ Significantly lower percentage or amount per cell in inflammation-stimulated AT-MSCs compared to the consecutive passage (*p*-value < 0.05).

Within the PM of AT-MSCs under normal conditions, a high percentage of cells constitutively expressed the NK ligands CD112 and CD155. In contrast, only a low percentage of AT-MSCs expressed UL16 binding protein3 (ULBP-3). In addition, a significant decrease in the percentage of AT-MSCs expressing these three markers was observed during the in vitro expansion. This was also the case for CD155 and ULBP-3 primed with a pro-inflammatory cocktail. In the presence of inflammation, a significantly higher amount of AT-MSCs expressed CD112. However, no significant differences in protein expression per cell could be observed for all three markers (Figure 6; Supplementary Tables S2 and S3).

## 4. Discussion

Stem-cell-based therapy has been the aim of deep investigation, leading to new hopes in the field of medicine. Mesenchymal stromal cells (MSCs) are progenitors’ cells with important biological properties and are thus considered to be a promising new strategy for disease management [1]. In presence of injury, MSCs may have to proliferate and to face inflammatory signals. Safe and efficient clinical applications require repeated administrations of a large amounts of cells (enrichment), with a well-established identify and responsive profile [19]. The communication of stem cells with their environment is an essential element in the plasticity of their properties. In line, inflammation is likely to control hematopoietic stem cell fate in health and disease conditions. Inflammatory signaling is deeply associated with the function and maintenance of the blood and other tissues, by regulating fate decisions at the stem cell level [20]. Thus, understanding the influence of inflammation and tissue-resident progenitors’ cells is an important issue [21]. Monitoring the inflammatory status of the patients at the time of MSC injection is required to improve the therapeutic effect [22].

Cellular therapy implies the investigation of the immunological profile and the immunomodulatory properties of the concerned cell population to guarantee the safety of the patient. This information is also indispensable in order to increase the success rate of the cell-based therapy by minimizing host rejection [23,24]. Since tissue or organ damage often goes hand-in-hand with inflammation, the behavior of the cells to be transplanted must be acquainted in an inflammatory environment before cell-based therapy can take place [25,26,27]. Due to their immunomodulatory and low immunogenicity profile, MSCs are generally recognized as an attractive therapeutic cell product [28]. To be suitable as a proper tissue source for cell-based therapy, some criteria need to be fulfilled. As such, MSCs need to be harvested in large amounts by utilization of a minimal invasive technique. Furthermore, the isolated MSCs must show high expansion potential and a specific panel of immune-related markers that are necessary for transplantation [7]. The tissue origin of MSCs, as well as their harvesting procedures, is also critical in determining their therapeutic potential. Thus, standardization of MSC isolation and culture methods is highly recommended [3,5,29,30,31,32]. Historically, bone marrow is the best known and characterized source of MSCs. However, the isolation of bone-marrow-derived MSCs (BM-MSCs) is invasive and the yield of harvested BM-MSCs remains low. Therefore, alternative sources have been proposed. Among them, adipose tissue forms a possible alternative for bone marrow, since it is ubiquitously present in every human, and high amount of MSCs can be obtained by liposuction, which is less invasive than bone marrow aspiration. On top of that, the lipoaspirate is considered to be medical waste, making adipose tissue an ideal source of MSCs [6,7,13]. Since the number of clinical applications using AT-MSCs is increasing, it is of main importance that the safety and full definition of this cell population is addressed [4,33,34,35]. In vitro expansion is of relevance to allow suitable amount of AT-MSCs for transplantation. It is clear from the literature that this in vitro expansion of MSCs can lead to distinct changes in MSCs phenotype [33], so defining expansion and environmental conditions, which are important for the biology of the cells, have to be considered when these cells are intended to be used for clinical scale-up [36].

In this study, AT-MSCs, obtained by the Ficoll gradient centrifugation method, were characterized concerning their immunophenotypical properties, when cultivated under normal and inflammatory conditions. Furthermore, the effect of cell expansion was examined. For this purpose, changes in the immunophenotype of AT-MSCs were determined by using flow cytometric analysis.

First of all, we found that the protein expression of HLA-ABC, CD40, CD54, CD106, CD274 and CD112 is significantly upregulated by AT-MSCs in a pro-inflammatory environment, whereas CD34, HLA-DR, HLA-G, CD40, CD62L, CD106, CD166, CD200, HO-1, CD155, and ULBP-3 expression is significantly altered during in vitro expansion.

For long time, CD34 was specified as a hematopoietic stem and progenitor cells marker, but evidence of its expression on BM-MSCs was indicated in the literature. Nevertheless, CD34 expression decreases to get totally lost in higher passages [6]. Due to these facts, CD34 expression is almost ubiquitously related to hematopoietic cells. The prevailing school of thought states that MSCs do not express CD34. However, robust data highlight a CD34 expression not only by MSCs but by several other non-hematopoietic cell types [37]. The CD34 positive cells may define a specific subset of progenitor cells with enhanced adhesive and homing properties, which are likely to mimic lymphocyte homing to the inflammatory sites [38,39].

Human leukocyte antigens (e.g., HLA-ABC and HLA-DR) and co-stimulatory molecules (e.g., CD40) play an important role in GVHD and allograft rejection, since they operate as targets and activators, respectively, of the immune system [11,14,15]. We found that HLA-DR expression in AT-MSCs significantly decreases during in vitro expansion, suggesting that the culture is becoming purer and therefore less immunogenic. Our findings are in accordance with previous flow cytometry studies that detected poor to negative expression of HLA-DR on expanded BM-MSCs [40]. Similarly, another study reported a very week level of HLA-DR mRNA expression on AT-MSCs, at only early passages (P2) [11]. Expansion rather than inflammation have decreased the expression of HLA-G in AT-MSCs. In contrast, we have recently demonstrated that hepatocytes and hepatogenic differentiated adult-derived human liver stem/progenitor cells (ADHLSCs) showed constitutive HLA-G expression that increased after the third step of differentiation. Both cell types were capable of inhibiting the proliferative response of PBMCs at least in part, through HLA-G [41].

CD40 is a transmembrane receptor of the TNF superfamily and expressed on a variety of antigen presenting cells (APCs). It binds to its ligand CD40L in order to facilitate the immune response [42]. Nevertheless, an increase of CD40 alone is not enough to evoke a complete T-lymphocyte activation, since a number of other co-stimulatory molecules are necessary [43]. Even upon inflammatory stimulation of AT-MSCs, no significant expression was observed of other co-stimulatory molecules necessary for an immune-response activation, including CD80, CD86 and CD134.

We and others have reported that AT-MSCs express a large panel of CAMs, enabling them to interact with activated immune cells and to respond to inflammatory signals [44,45]. These cellular interactions are important for their immunosuppressive functioning. Therefore, changes in the expression of CAMs, such as CD54 (ICAM1), CD62L (LECAM1), CD106 (VCAM1) and CD166 (ALCAM), could have a significant impact on their immunomodulatory properties. Similar upregulation of CAMs has been reported on MCSs from other tissue source, such as WJ-MSCs after stimulation inflammatory cytokines [45]. The passage number in normal conditions seems to affect differently the expression of other CAM marker in a cell-type-dependent manner. While our study showed a decreased percentage of cells expressing CD29 and CD44, almost 100% of human MSCs derived from bone marrow expressed these markers, regardless of passage number, but expression of CD106 was somehow variable [24,46].

Immunomodulation is also regulated by the expression of regulatory factors such as HO-1 and CD274, of which we found that the expression is affected by in vitro expansion and inflammation, respectively. HO-1 is known as antioxidant, cytoprotective and anti-inflammatory enzyme. HO-1 may act by decreasing TNFα levels in parallel to a significant increase of IL-10 [47]. As such, a decreased HO-1 expression during in vitro expansion could therefore lead to a reduced immunomodulatory capacity of AT-MSCs. Although continuous passaging of AT-MSCs has been reported to decrease the immunosuppressive capabilities of MSCs, findings suggest that AT-MCSs may represent an advantageous immunosuppressive agent over BM-MSCs and a preferred source for regenerative medicine application [11].

CD274, also referred to as programmed death ligand 1 (PD-L1), is suggested to hamper an immune response. Binding of the PD-L1 molecule to the PD-1 receptor will downregulate T-lymphocyte proliferation and cytokine secretion [48]. Hence, its increased expression in AT-MSCs in a pro-inflammatory environment suggests an upregulated immunosuppressive capacity. Inflammatory stimuli (reviewed in [49]) has been shown to regulate the functions of MSCs by modulating their phenotype and secretome.

Finally, NK cells play an important role in both the innate and adaptive immunity against the allograft, since they can recognize autologous and allogeneic objects and are able of killing targets. Cross-interaction and modulation are occurring during NK cells and AT-MSCs co-culture. In the presence of AT-MSCs, the expression profile of ligands and receptors relevant for NK cell biology was significantly modulated. IFN-γ and TNF-α secretions were significantly increased, while the proliferation of NK cells was slightly diminished [50]. For this purpose, the NK cells are endowed with activating receptors that will bind specific NK ligands, allowing cell activation [51]. In this study, we report that inflammation enhances the expression of the NK ligand CD112 on AT-MSCS. This finding suggests a possible increased NK-mediated cytotoxity against AT-MSCs, especially in a pro-inflammatory environment, and consequently their elimination upon transplantation. This NK-mediated lysis will also trigger the production of cytokines. Nevertheless, NK-mediated lysis of AT-MSCs in a pro-inflammatory environment might be inhibited to a certain extent as a result of the upregulation of HLA class I molecules (HLA-ABC) [52]. Furthermore, we observed that in vitro expansion downregulates the expression of the NK ligands CD155 and ULBP-3 and thus potentially makes them more tolerant toward NK cells. Further investigations in the influence of inflammatory signals, which fluctuate considerably during tissue damage, on the immuno-biology of MSCs will accelerate the development of promising cell-therapy strategies [53]. It is now recognized that the communication of AT-MSCs with the environment is an essential part of their tissue-repair process. MSCs can actively sense their surroundings and modulate, accordingly, their fate and behavior. In presence of injury, AT-MSCs may have to proliferate (cell-expansion), as well as respond to inflammatory signals (inflammation), in order to create an immuno-reparative microenvironment. It is therefore important to understand the effect of cell-expansion and inflammation on the immune-biology of MSCs, in order to maximize their beneficial outcomes. In comparison to previous studies, this is the first time that these two relevant features of MSCs are explored and, most importantly, investigated in combination. The immunological profile of the AT-MSCs, obtained by means of the standard Ficoll gradient centrifugation was characterized by flow cytometric analyses, under these two conditions. The expression of cell adhesion molecules, immune regulatory molecules and natural killer ligands were thus investigated. A detailed characterization of AT-MSCs under these conditions, with 28 examined markers, is therefore presented. Several changes and alterations within the expression profiles of these molecules are observed during cell-expansion and inflammation. Such an analysis will greatly help to understand the molecular mechanisms that may improve or hamper the therapeutic potential of AT-MSCs, contributing thus to the efforts in developing a quality, safe and efficient cellular-therapeutic product.

## Supplementary Materials

The following are available online at https://www.mdpi.com/2077-0383/9/3/696/s1. Table S1: List of antibodies, Table S2: Percentage (%) of positive cells for each marker, Table S3: Mean fluorescence intensity (MFI) of each marker

## Abbreviations

ALCAM	activated leukocyte cell adhesion molecule
APC	antigen presenting cell
AT	adipose tissue
AT-MSCs	adipose tissue-derived mesenchymal stromal cells
BM-MSCs	bone marrow-derived mesenchymal stromal cells
BSA	bovine serum albumin
CAM	cell adhesion molecule
CD	cluster of differentiation
GVHD	graft-versus-host disease
HLA	human leukocyte antigen
HO	heme oxygenase
i	intracellular
ICAM	intercellular adhesion molecule
IFN	interferon
IL	interleukin
LECAM	leukocyte endothelial cell adhesion molecule
m	membrane bound
MFI	mean fluorescence intensity
MSCs	mesenchymal stromal cells
NK	natural killer
P	passage
PBS	phosphate-buffered saline
PD-L	programmed death ligand
PM	primary culture
SD	standard deviation
SEM	standard error of the mean
TNF	tumor necrosis factor
ULBP	UL16 binding protein
VCAM	vascular cell adhesion molecule
