# Supplementary material for: The Impact of Cell-Expansion and Inflammation on The Immune-Biology of Human Adipose Tissue-Derived Mesenchymal Stromal Cells
Source: J Clin Med. 2020 Mar 4;9(3):696. doi: 10.3390/jcm9030696 (PMC7141238; doi:10.3390/jcm9030696)
Supplement: Supplementary file 1 [file jcm-09-00696-s001.pdf]

TABLE S1 : List of antibodies

Primary antibody	Order ID	Species	Dilution	Source
**Endothelial and stromal markers**				
anti-CD34-PC5	555823	mouse	1/20	BD
anti-CD105-FITC	326040	mouse	1/20	AC
**Human leukocyte antigens**				
anti-HLA-ABC-PE-Cy5	15998342	mouse	1/20	EB
anti-HLA-DR-PerCP	347402	mouse	1/20	BD
anti-HLA-G1-PE	1P292C100	mouse	1/20	ExBIO
**Immune co-stimulatory molecules**				
anti-CD40-PE	130094135	mouse	1/20	MB
anti-CD80-FITC	11080942	mouse	1/20	EB
anti-CD86-APC	130094876	mouse	1/20	MB
anti-CD252-PE	326308	mouse	1/20	BL
anti-CD134-FITC	350006	mouse	1/20	BL
**Cell adhesion molecules**				
anti-CD29-PE-Cy5	559882	mouse	1/20	BD
anti-CD44-FITC	130095195	mouse	1/20	MB
anti-CD49e-PE	555617	mouse	1/20	BD
anti-CD54-PE	555511	mouse	1/20	BD
anti-CD58-FITC	555920	mouse	1/20	BD
anti-CD62L-Fluorescein	BBA21	mouse	1/20	R&D
anti-CD102-FITC	328507	mouse	1/20	BL
anti-CD106-PE-Cy5	551148	mouse	1/20	BD
anti-CD146-PC5	A22364	mouse	1/20	BC
anti-CD166-PE	559263	mouse	1/20	BD
**Immune regulatory molecules**				
anti-CD39-FITC	328205	mouse	1/20	BL
anti-CD73-PE	344003	mouse	1/20	BD
anti-CD200-APC	329208	mouse	1/20	BL
anti-CD274-PE	557924	mouse	1/20	BD
anti-HO-1-PE	ADI-OSA-111	mouse	1/20	ELS
**NK ligands**				
anti-CD112-PE	337410	mouse	1/20	BL
anti-CD155-PE	337508	mouse	1/20	BL
anti-ULBP-3-PE	FAB1517P	mouse	1/20	R&D

TABLE S2 : Percentage (%) of positive cells for each marker

Marker/Culture passage	PM		P1		P2		P3		P4	
	Constitutive	*Inflammation*	*Constitutive*	*Inflammation*	*Constitutive*	*Inflammation*	*Constitutive*	*Inflammation*	*Constitutive*	*Inflammation*
Endothelial and stromal markers										
CD34	19,00 ± 1,06	17,83 ± 1,08	8,83 ± 0,60	7,33 ± 1,31	3,33 ± 0,61	3,00 ± 0,52	1,67 ± 0,49	1,83 ± 0,40	1,17 ± 0,17	1,00 ± 0,00
CD73	95,00 ± 0,36	80,50 ± 15,11	97,17 ± 0,75	96,17 ± 1,89	97,50 ± 0,43	95,67 ± 1,23	97,50 ± 0,67	98,17 ± 0,31	97,00 ± 0.73	97.50 ± 0.43
CD105	85,33 ± 4,39	66,00 ± 13,89	92,83 ± 2,21	73,50 ± 10,35	91,83 ± 4,04	74,00 ± 10,70	87,33 ± 1,86	75,83 ± 9,53	78,17 ± 3,16	57,33 ± 10,78
Human leukocyte antigens										
HLA-ABC	99,00 ± 0,00	98,83 ± 0,31	98,50 ± 0,34	98,33 ± 0,42	97,00 ± 0,52	96,67 ± 1,02	97,00 ± 0,58	95,33 ± 1,28	95,83 ± 0,60	95,00 ± 1,12
HLA-DR	5,50 ± 0,67	5,83 ± 1,25	3,33 ± 0,42	2,17 ± 0,40	2,50 ± 0,22	2,00 ± 0,36	1,33 ± 0,21	1,00 ± 0,00	1,00 ± 0,00	1,33 ± 0,21
mHLA-G	15,50 ± 1,18	19,33 ± 1,20	11,33 ± 0,99	13,00 ± 0,73	6,83 ± 0,83	7,17 ± 1,20	3,00 ± 0,52	3,33 ± 0,92	1,33 ± 0,33	1,33 ± 0,21
iHLA-G	84,67 ± 1,26	84,00 ± 1,29	79,33 ± 1,36	80,67 ± 1,71	76,00 ± 1,92	77,17 ± 1,54	78,83 ± 1,54	74,33 ± 1,08	70,67 ± 1,69	70,67 ± 0,61
Co-stimulatory molecules										
CD40	53,17 ± 7,72	90,67 ± 1,58	23,00 ± 3,24	73,50 ± 8,03	17,17 ± 2,02	70,67 ± 5,30	11,50 ± 1,89	68,50 ± 4,79	9,33 ± 1,73	69,17 ± 6,07
CD80	3,00 ± 0,36	2,33 ± 0,42	2,00 ± 0,00	1,83 ± 0,17	1,67 ± 0,21	1,83 ± 0,17	1,00 ± 0,00	1,33 ± 0,21	1,00 ± 0,00	1,17 ± 0,17
CD86	8,33 ± 0,95	9,67 ± 1,94	4,00 ± 0,36	4,33 ± 0,42	3,17 ± 0,31	5,17 ± 1,25	2,33 ± 0,21	4,83 ± 2,06	1,67 ± 0,33	4,67 ± 1,56
CD134	4,17 ± 1,08	4,33 ± 1,23	2,33 ± 0,42	3,50 ± 0,81	3,00 ± 0,26	3,17 ± 0,40	2,17 ± 0,31	2,33 ± 0,42	1,00 ± 0,00	1,67 ± 0,33
CD252	14,17 ± 2,18	41,50 ± 15,81	7,83 ± 1,68	23,67 ± 4,59	6,50 ± 1,34	20,33 ± 3,58	5,17 ± 1,01	11,00 ± 2,65	2,50 ± 0,50	7,33 ± 2,32
Cell adhesion molecules										
CD29	97.67 ± 0.56	95.83 ± 0.87	98.33 ± 0.42	97,83 ± 0,65	96,17 ± 0,54	96,50 ± 0,92	86,00 ± 3,45	87,67 ± 3,04	74,00 ± 3,60	74,17 ± 4,91
CD44	98,50 ± 0,34	97,50 ± 0,67	98,00 ± 0,36	97,33 ± 0,49	98,00 ± 0,26	96,50 ± 0,76	96,50 ± 0,67	96,67 ± 0,61	91,83 ± 1,08	94,33 ± 1,69
CD49e	97,83 ± 0,17	97,00 ± 0,45	98,00 ± 0,45	97,50 ± 0,62	97,33 ± 0,67	94,83 ± 2,04	95,00 ± 0,86	94,33 ± 0,92	92,00 ± 1,93	91,83 ± 1,97
CD54	42,83 ± 3,00	96,00 ± 0,52	53,00 ± 2,21	93,83 ± 2,10	59,50 ± 1,59	96,00 ± 0,58	68,67 ± 2,08	96,50 ± 1,02	74,00 ± 2,07	97,50 ± 0,96
CD58	26,50 ± 1,93	57,67 ± 8,54	14,67 ± 1,20	38,67 ± 4,63	11,00 ± 1,16	27,00 ± 5,26	5,83 ± 1,35	13,50 ± 1,80	2,17 ± 0,65	8,33 ± 1,67
CD62L	12,00 ± 0,93	12,67 ± 1,50	7,83 ± 0,60	7,50 ± 1,38	3,83 ± 0,48	4,33 ± 0,61	2,17 ± 0,17	2,67 ± 0,61	1,17 ± 0,17	2,17 ± 0,65
CD102	3,50 ± 0,43	3,17 ± 0,79	2,33 ± 0,33	3,00 ± 0,36	1,67 ± 0,21	2,00 ± 0,26	1,17 ± 0,17	1,50 ± 0,22	1,00 ± 0,00	1,67 ± 0,33
CD106	2,50 ± 0,56	29,17 ± 0,60	2,67 ± 0,33	32,67 ± 2,17	2,17 ± 0,31	27,33 ± 2,75	2,00 ± 0,26	19,50 ± 1,26	1,17 ± 0,17	18,00 ± 0,58
CD146	15,17 ± 0,79	15,83 ± 2,26	11,50 ± 1,48	12,17 ± 2,24	9,33 ± 1,43	10,50 ± 1,96	6,50 ± 0,96	5,00 ± 0,86	3,33 ± 0,56	2,83 ± 0,60
CD166	94,67 ± 1,20	94,33 ± 1,67	88,67 ± 1,91	85,67 ± 3,10	83,33 ± 1,94	82,33 ± 3,55	77,67 ± 2,79	76,17 ± 3,20	64,67 ± 3,94	66,67 ± 4,10
Immunoregulatory molecules										
CD39	2,17 ± 0,31	1,33 ± 0,21	1,33 ± 0,21	1,17 ± 0,17	1,33 ± 0,21	1,17 ± 0,17	1,00 ± 0,00	1,17 ± 0,17	1,00 ± 0,00	1,00 ± 0,00
CD200	34,33 ± 1,89	35,33 ± 1,94	21,50 ± 0,76	21,67 ± 0,95	14,67 ± 1,05	15,00 ± 0,86	7,00 ± 0,58	7,83 ± 0,87	4,17 ± 0,79	3,50 ± 0,43
CD274	23,67 ± 0,67	93,00 ± 1,06	15,17 ± 1,14	93,67 ± 1,12	7,50 ± 0,43	89,17 ± 2,52	4,67 ± 0,42	89,83 ± 0,94	3,67 ± 0,49	88,17 ± 4,09
HO-1	93,00 ± 1,26	93,50 ± 1,06	89,83 ± 1,11	90,17 ± 1,14	86,50 ± 1,56	85,83 ± 0,91	81,33 ± 1,56	83,50 ± 1,20	75,67 ± 2,04	76,67 ± 1,54
Natural killer ligands										
CD112	86,33 ± 1,45	92,00 ± 1,65	79,67 ± 1,86	84,17 ± 5,88	57,83 ± 3,23	76,50 ± 6,57	48,83 ± 2,10	72,67 ± 4,64	42,83 ± 2,87	65,00 ± 5,69
CD155	90,33 ± 2,32	88,33 ± 2,51	81,83 ± 1,40	81,33 ± 1,58	77,67 ± 1,05	77,00 ± 1,83	70,33 ± 0,99	68,00 ± 1,67	62,33 ± 1,23	60,67 ± 2,19
ULBP3	4,33 ± 0,67	4,33 ± 0,56	2,83 ± 0,31	2,33 ± 0,21	2,17 ± 0,17	2,00 ± 0,26	1,50 ± 0,22	2,17 ± 0,48	1,17 ± 0,17	1,17 ± 0,17

TABLE S3 : Mean fluorescence intensity (MFI) of each marker

Marker/Culture passage	PM		P1		P2		P3		P4	
	Constitutive	*Inflammation*	*Constitutive*	*Inflammation*	*Constitutive*	*Inflammation*	*Constitutive*	*Inflammation*	*Constitutive*	*Inflammation*
Endothelial and stromal markers										
CD34	21,33 ± 2,78	23,33 ± 3,16	33,50 ± 4,70	34,50 ± 5,58	42,17 ± 8,60	44,50 ± 8,27	36,83 ± 2,43	36,33 ± 3,77	40,50 ± 6,35	41,50 ± 5,04
CD73	92,33 ± 31,53	136,20 ± 23,34	107,70 ± 20,32	131,20 ± 14,67	103,20 ± 17,16	130,20 ± 12,69	128,50 ± 19,87	131,70 ± 16,71	182,50 ± 31,72	176,30 ± 23,87
CD105	61,50 ± 4,72	55,83 ± 7,05	59,00 ± 10.04	48.67 ± 7.94	82.00 ± 18.60	57.00 ± 9.69	75.67 ± 11.51	61.50 ± 7.62	79.50 ± 5.44	70.00 ± 4.99
Human leukocyte antigens										
HLA-ABC	185.70 ± 42.88	417.00 ± 80.96	123.30 ± 19.47	314.30 ± 32.22	91.33 ± 9.09	262.20 ± 25.81	83.83 ± 8.85	208.50 ± 24.10	88.67 ± 5.43	226.80 ± 27.92
HLA-DR	58.17 ± 8.11	69.67 ± 7.52	20.83 ± 2.33	24.83 ± 4.96	21.67 ± 2.93	21.83 ± 3.05	22.33 ± 2.62	21.33 ± 2.98	24.00 ± 2.25	23.33 ± 3.05
mHLA-G	40,83 ± 7,12	37,17 ± 7,01	33,17 ± 7,27	34,67 ± 7,53	32,83 ± 6,75	35,33 ± 7,05	35,00 ± 6,14	35,83 ± 6,51	41,83 ± 6,19	41,00 ± 7.24
iHLA-G	21,83 ± 5,22	27,00 ± 6,06	28,00 ± 5,43	28,50 ± 6,03	27,00 ± 3,47	30,33 ± 5,32	31,67 ± 2,12	33,33 ± 4,10	38,83 ± 3,08	38,00 ± 2,93
Co-stimulatory molecules										
CD40	26,33 ± 4,05	80,67 ± 17,59	28,83 ± 4,81	49,50 ± 6,26	36,00 ± 4,62	57,83 ± 5,55	37,00 ± 4,09	53,67 ± 7,52	42,17 ± 2,57	63,00 ± 9,70
CD80	20,00 ± 1,53	27,33 ± 3,90	47,50 ± 12,82	55,33 ± 13,96	38,00 ± 5,56	42,00 ± 6,05	39,33 ± 3,04	34,17 ± 2,70	48,50 ± 6,14	51,33 ± 12,32
CD86	78,83 ± 19,97	105,20 ± 21,70	38,50 ± 8,32	72,17 ± 8,44	66,00 ± 15,03	63,83 ± 20,23	73,00 ± 30,37	66,33 ± 13,86	49,17 ± 18,15	74,80 ± 19,59
CD134	21,83 ± 2,59	29,83 ± 3,15	47,67 ± 11,19	53,67 ± 16,05	35,50 ± 4.81	40.17 ± 6.14	37.67 ± 2.44	35.50 ± 1.48	56.00 ± 12.02	41.67 ± 4.36
CD252	39,33 ± 7,85	44,00 ± 7,05	31,67 ± 6,38	29,00 ± 6,05	28,17 ± 3,46	25,00 ± 4,91	36,33 ± 2,12	29,17 ± 2,04	48,83 ± 6,87	38,17 ± 7,45
Cell adhesion molecules										
CD29	48,67 ± 9,60	44,67 ± 5,54	95,17 ± 11,06	79,83 ± 11,26	83,33 ± 12,21	82,67 ± 14,42	49,33 ± 10,39	42,83 ± 9,82	47,17 ± 10,69	44,17 ± 10,68
CD44	158,50 ± 31,08	166,50 ± 39,66	144,50 ± 16,62	162,00 ± 15,08	134,80 ± 9,56	153,30 ± 12,51	125,00 ± 8.21	152.50 ± 12.85	139.50 ± 10.62	164,00 ± 13,86
CD49e	108,20 ± 22,72	82,67 ± 11,45	96,67 ± 13,42	79,00 ± 7,96	91,17 ± 12,26	87,83 ± 11,45	96,00 ± 18,82	89,67 ± 18,26	97,67 ± 16,51	91,17 ± 11,68
CD54	51,50 ± 11,60	534,20 ± 118,00	46,00 ± 10,07	455,20 ± 48,51	40,33 ± 4,60	531,00 ± 97,57	52,33 ± 7,27	446,70 ± 35,70	70,17 ± 8,60	476,30 ± 56,98
CD58	26,17 ± 5,41	31,00 ± 6,70	37,50 ± 7,69	38,67 ± 9,50	42,00 ± 8,84	43,50 ± 9,61	44,50 ± 7,04	37,67 ± 8,03	48,17 ± 7,21	43,00 ± 6,60
CD62L	27,83 ± 2,75	31,33 ± 4,30	37,33 ± 7,19	37,50 ± 7,35	36,00 ± 4,65	41,17 ± 8,06	48,17 ± 8,69	48,00 ± 8,89	48,83 ± 7,01	43,00 ± 6,99
CD102	25,50 ± 4,02	31,17 ± 6,88	26,50 ± 3,68	28,83 ± 3,54	38,83 ± 3,16	42,17 ± 4,59	35,33 ± 5,14	33,33 ± 2,59	53,50 ± 13,96	56,17 ± 11,30
CD106	23,00 ± 3,17	21,67 ± 0,95	19,50 ± 2,14	22,17 ± 1,85	19,00 ± 2,90	22,50 ± 2,03	19,83 ± 2,17	22,00 ± 0,86	18,50 ± 1,38	23,00 ± 2,17
CD146	26,50 ± 2.47	27.33 ± 2,64	22,00 ± 3,33	23,33 ± 2,65	26,67 ± 2,50	25,33 ± 1,67	25,33 ± 2,86	26,33 ± 2,78	26,83 ± 2,40	25,00 ± 2,58
CD166	56,67 ± 7,44	63,50 ± 11,71	59,33 ± 7,45	50,83 ± 6,50	49,17 ± 4,47	47,17 ± 3,72	54,17 ± 7,46	51,00 ± 7,50	38,50 ± 5,93	38,17 ± 5,87
Immunoregulatory molecules										
CD39	22,33 ± 2,50	32,33 ± 5,28	41,83 ± 10,90	41,67 ± 11,06	40,67 ± 8,81	41,67 ± 9,46	33,83 ± 2,93	31,17 ± 1,40	41,17 ± 4,43	42,17 ± 3,94
CD200	24,17 ± 2,50	23,00 ± 3,11	27,17 ± 4,60	29,00 ± 5,17	26,67 ± 4,44	30,00 ± 4,25	24,83 ± 3,91	26,50 ± 4,08	32,17 ± 1,17	32,33 ± 2,44
CD274	27,67 ± 8,04	59,50 ± 10,18	24,33 ± 4,52	67,17 ± 11,77	26,00 ± 3,12	66,67 ± 8,63	26,67 ± 3,10	62,83 ± 9,27	37,67 ± 3,86	85,00 ± 6,83
HO-1	32,33 ± 8,14	33,17 ± 7,64	37,00 ± 8,54	40,50 ± 8,35	31,33 ± 1,69	35,17 ± 2,46	33,33 ± 1,84	32,00 ± 2,21	33,67 ± 1,31	32,67 ± 1,76
Natural killer ligands										
CD112	34,00 ± 4,94	48,00 ± 9,71	44,67 ± 14,97	57,67 ± 13,69	39,00 ± 6,18	45,83 ± 5,71	32,00 ± 3,31	32,50 ± 2,66	38,67 ± 5,35	41,33 ± 5,36
CD155	37,67 ± 5,48	33,17 ± 3,83	48,33 ± 9,14	51,17 ± 10,09	53,67 ± 8,59	53,33 ± 7,24	54,67 ± 7,60	56,83 ± 6,91	37,83 ± 6,07	38,33 ± 5,91
ULBP3	27,67 ± 4,42	32,00 ± 6,37	30,17 ± 5,25	31,00 ± 5,12	37,17 ± 7,42	35,50 ± 5,19	33,00 ± 5,54	30,33 ± 3,76	41,83 ± 5,42	39,50 ± 4,79
